# Circadian Gene Variants and Susceptibility to Type 2 Diabetes: A Pilot Study

**DOI:** 10.1371/journal.pone.0032670

**Published:** 2012-04-02

**Authors:** M. Ann Kelly, Simon D. Rees, M. Zafar I. Hydrie, A. Samad Shera, Srikanth Bellary, J. Paul O’Hare, Sudhesh Kumar, Shahrad Taheri, Abdul Basit, Anthony H. Barnett, DIAGRAM consortium, SAT2D consortium

**Affiliations:** 1 College of Medical and Dental Sciences, University of Birmingham, Birmingham, United Kingdom; 2 BioMedical Research Centre, Heart of England NHS Foundation Trust, Birmingham, United Kingdom; 3 Baqai Institute of Diabetology and Endocrinology (BIDE), Karachi, Pakistan; 4 Diabetic Association of Pakistan, Karachi, Pakistan; 5 School of Life and Health Sciences, Aston University, Birmingham, United Kingdom; 6 Warwick Medical School, University of Warwick, Coventry, United Kingdom; IPO, Inst Port Oncology, Portugal

## Abstract

**Background:**

Disruption of endogenous circadian rhythms has been shown to increase the risk of developing type 2 diabetes, suggesting that circadian genes might play a role in determining disease susceptibility. We present the results of a pilot study investigating the association between type 2 diabetes and selected single nucleotide polymorphisms (SNPs) in/near nine circadian genes. The variants were chosen based on their previously reported association with prostate cancer, a disease that has been suggested to have a genetic link with type 2 diabetes through a number of shared inherited risk determinants.

**Methodology/Principal Findings:**

The pilot study was performed using two genetically homogeneous Punjabi cohorts, one resident in the United Kingdom and one indigenous to Pakistan. Subjects with (N = 1732) and without (N = 1780) type 2 diabetes were genotyped for thirteen circadian variants using a competitive allele-specific polymerase chain reaction method. Associations between the SNPs and type 2 diabetes were investigated using logistic regression. The results were also combined with *in silico* data from other South Asian datasets (SAT2D consortium) and white European cohorts (DIAGRAM+) using meta-analysis. The rs7602358G allele near *PER2* was negatively associated with type 2 diabetes in our Punjabi cohorts (combined odds ratio [OR] = 0.75 [0.66–0.86], p = 3.18×10^−5^), while the *BMAL1* rs11022775T allele was associated with an increased risk of the disease (combined OR = 1.22 [1.07–1.39], p = 0.003). Neither of these associations was replicated in the SAT2D or DIAGRAM+ datasets, however. Meta-analysis of all the cohorts identified disease associations with two variants, rs2292912 in *CRY2* and rs12315175 near *CRY1,* although statistical significance was nominal (combined OR = 1.05 [1.01–1.08], p = 0.008 and OR = 0.95 [0.91–0.99], p = 0.015 respectively).

**Conclusions/significance:**

None of the selected circadian gene variants was associated with type 2 diabetes with study-wide significance after meta-analysis. The nominal association observed with the *CRY2* SNP, however, complements previous findings and confirms a role for this locus in disease susceptibility.

## Introduction

Accumulating evidence suggests that dysregulation of the circadian clock plays an important role in glucose homeostasis and the development of type 2 diabetes. Cross-sectional and prospective studies have shown that voluntary sleep curtailment to 6 hours or less per day is associated with increased fasting glucose levels, hyperinsulinaemia and reduced insulin sensitivity, leading to an increased risk of diabetes [1], [2]. Laboratory-imposed circadian desynchrony, resulting in misalignment between behavioural cycles (such as sleep/wake and fasting/feeding) and endogenous circadian rhythm, has also been shown to result in increased postprandial glucose and insulin levels, increased mean arterial blood pressure and decreased leptin levels [3]. These metabolic disturbances could explain the increased risk of diabetes, obesity and cardiovascular disease observed in shift-workers [4], [5].

Circadian rhythms are controlled and maintained by transcription-translation-based networks of positive and negative feedback loops that oscillate with 24-hour rhythmicity [6]. A recent study in mice showed that mutations in the genes encoding the CLOCK and BMAL1 transcription factors resulted in impaired glucose tolerance, reduced insulin secretion and defects in islet development, while ablation of the endogenous pancreatic clock resulted in the development of diabetes [7]. Variants of circadian genes, such as *MTNR1B*, *CRY2*, *PER3*, *PER2* and *BMAL1*, have recently been implicated as determinants of fasting glucose levels and/or diabetes risk in humans [8]–[12], while single nucleotide polymorphisms (SNPs) in the *CLOCK* and *PER2* genes were reported to be associated with measures of obesity [13,14]. Further investigations of circadian genes as risk markers for metabolic disease are therefore warranted.

We aimed to investigate the role of circadian gene variants as susceptibility determinants for type 2 diabetes. Before embarking on a comprehensive tag SNP analysis, however, we carried out a pilot study of selected variants chosen on the basis of their previously reported association with prostate cancer, another condition where risk has been shown to be modified by circadian misalignment [15]. The relationship between type 2 diabetes and prostate cancer is controversial. Although a number of studies have suggested that diabetic men appear to be less prone to prostate cancer than non-diabetic men (relative risk = 0.84 from a recent meta-analysis [16]), this negative correlation between the diseases has not been observed in all populations. Despite this epidemiological inconsistency, there is compelling evidence of a genetic link between the two diseases. Variants in *HNF1B*, *UCP2*, *SLC2A2*, *IGF2BP2*, *TCF7L2* and *CAPN10* have been shown to predispose to type 2 diabetes and protect against prostate cancer or vice versa [17], [18]. A recent study also reported an inverse association between the risk of prostate cancer and a genetic risk score for type 2 diabetes [19], although a subsequent study of a subset of 17 diabetes-associated SNPs in a multiethnic cohort found no evidence of an impact on prostate cancer risk [20]. The *JAZF1* gene has been implicated in both disorders, although the cancer-protective and diabetes-predisposing effects are mediated by different SNPs [17]. Similarly, independent association signals for the two diseases have been identified in the *THADA* gene by genome-wide association studies (GWAS) [21], [22]. A recent report by Zhu *et al* [23] suggested that twelve variants in, or close to, nine circadian-related genes were associated with overall prostate cancer risk, or risk of more or less aggressive disease. The aim of our pilot study was to determine whether these SNPs also influence the risk of type 2 diabetes.

## Materials and Methods

### Ethics Statement

Informed written consent was obtained from all participants and the study was approved by the Birmingham East, North and Solihull Research Ethics Committee (for participants resident in the United Kingdom) and the Baqai Institute of Diabetology and Endocrinology Institutional Review Board (for participants resident in Pakistan).

### Study Participants

The study was performed using two populations of South Asian origin. UK-resident subjects (892 with type 2 diabetes, 471 normoglycaemic individuals) were recruited from Birmingham and Coventry as part of the United Kingdom Asian Diabetes Study (UKADS) [24] (UKADS registered clinical trial number; ISRCTN38297969). Pakistan-based subjects (840 with type 2 diabetes, 1309 normoglycaemic individuals) were recruited from the Mirpur region of Azad Kashmir (Diabetes Genetics in Pakistan study, DGP). All individuals were of Punjabi ancestry and originated predominantly from Mirpur. Diagnosis of type 2 diabetes was established using World Health Organisation criteria [25]. Normoglycaemia was defined as random blood glucose <7mmol/l in the UKADS control subjects and fasting whole blood glucose ≤5.6mmol/l in the DGP control subjects. Details of the study subjects have been published previously [26].

### Genotyping

The subjects (N = 3512) were genotyped for selected circadian gene variants using the KASPar method (KBiosciences, Hoddesdon, UK). The twelve SNPs previously associated with prostate cancer were investigated (*CLOCK*, rs11133373; *BMAL1*, rs7950226; *PER1*, rs885747 and rs2289591; *PER2*, rs7602358; *PER3*, rs1012477; *CRY1*, rs12315175; *CRY2*, rs2292912; *CSNK1E*, rs1534891; *NPAS2*, rs1369481, rs895521 and rs17024926)[23]. In addition we also genotyped for *BMAL1* rs11022775, as this has previously been shown to be part of a susceptibility haplotype for type 2 diabetes along with rs7950226 [12]. For all SNPs, genotyping success rates were >96% and error rates in 384 duplicate samples were <0.6%.

### Statistical Analysis

Statistical analysis was performed using STATA IC (version 10.1)(Stata Corporation, College Station, TX, USA). Genotype frequencies for each SNP were checked for deviation from Hardy-Weinberg equilibrium in the normoglycaemic subjects using an exact test. The association between SNPs and type 2 diabetes was tested using logistic regression, adjusting for age and sex. The indigenous and migrant populations were analysed separately in the first instance; odds ratio (OR) values were then combined using inverse variance weighted meta-analysis, implemented in METAN. A study-wide significance threshold of p<0.0039 was applied to the analysis of the 13 SNPs in these datasets. To improve sample size and get a truer picture of the impact of the SNPs on disease risk, meta-analysis was used to combine data from the pilot study with summary statistics from the GWA phases of the expanded DIAbetes Genetics Replication And Meta-analysis (DIAGRAM+) study [27] and the South Asian Type 2 Diabetes (SAT2D) study [28]. The DIAGRAM+ cohort comprised 8,130 cases with type 2 diabetes and 38,987 control subjects of white European ancestry, while the SAT2D dataset consisted of 5,561 South Asian individuals with type 2 diabetes and 14,458 ethnically-matched control subjects. Heterogeneity of OR values between the UKADS and DGP study populations, and between the combined UKADS/DGP dataset, SAT2D and DIAGRAM+ cohorts, was assessed using Cochran’s Q statistic. Haplotype analysis for the *BMAL1* locus was performed using a logistic regression framework implemented in PLINK [29].

## Results

The clinical characteristics of the UKADS/DGP study subjects are shown in Table S1. None of the studied SNPs deviated significantly from Hardy-Weinberg equilibrium in the control groups from these cohorts after correction for the number of tests performed. Table 1 shows the OR values (with 95% confidence intervals) and significance values for the association of type 2 diabetes with each of the 13 studied SNPs in our South Asian populations, along with the DIAGRAM+ and SAT2D cohorts. OR values did not differ significantly between the indigenous and migrant Punjabi populations in our study; therefore results are presented for the combined UKADS/DGP dataset. Sex-specific analysis of this cohort showed no significant differences in OR values between males and females (Table S2).

**Table 1:** Odds ratios and p values for the association of circadian gene variants with type 2 diabetes.

			UKADS/DGP			SAT2D		DIAGRAM+		ALL DATASETS		
Gene region	SNP	Allele (minor/common)	MAF	OR (95% CI)	p	OR (95% CI)	p	OR (95% CI)	p	OR (95% CI)	p	p_het_
PER3	rs1012477	C/G	0.05	1.11 (0.89–1.39)	0.345	0.95 (0.85–1.06)	0.345	0.98 (0.93–1.04)	0.510	0.98 (0.94–1.03)	0.438	0.447
BMAL1	rs11022775	T/C	0.16	1.22 (1.07–1.39)	**0.003**	0.98 (0.93–1.04)	0.519	0.91 (0.83–0.99)	**0.044**	0.99 (0.94–1.04)	0.633	0.001
CLOCK	rs11133373	G/C	0.38	0.93 (0.84–1.03)	0.150	0.98 (0.92–1.04)	0.449	0.99 (0.95–1.04)	0.796	0.98 (0.95–1.01)	0.267	0.476
CRY1	rs12315175	C/T	0.07	0.94 (0.78–1.12)	0.471	0.92 (0.84–1.01)	0.089	0.96 (0.91–1.01)	0.085	0.95 (0.91–0.99)	**0.015**	0.794
NPAS2	rs1369481	T/C	0.24	0.94 (0.84–1.05)	0.264	1.10 (1.04–1.16)	**3.78×10^−4^**	0.99 (0.95–1.04)	0.780	1.03 (0.99–1.06)	0.102	0.003
CSNK1E	rs1534891	T/C	0.19	1.03 (0.91–1.17)	0.613	0.98 (0.92–1.03)	0.389	1.00 (0.95–1.06)	0.908	0.99 (0.96–1.03)	0.728	0.639
NPAS2	rs17024926	C/T	0.32	1.06 (0.96–1.18)	0.268	0.93 (0.89–0.98)	**0.007**	1.01 (0.97–1.05)	0.595	0.99 (0.96–1.02)	0.336	0.018
PER1	rs2289591	A/C	0.14	0.96 (0.83–1.11)	0.582	1.00 (0.93–1.09)	0.908	0.96 (0.92–1.01)	0.101	0.97 (0.93–1.01)	0.150	0.624
CRY2	rs2292912	C/G	0.27	1.02 (0.91–1.13)	0.752	1.05 (1.00–1.12)	0.057	1.05 (1.00–1.10)	0.056	1.05 (1.01–1.08)	**0.008**	0.846
PER2	rs7602358	G/T	0.16	0.75 (0.66–0.86)	**3.18×10^-5^**	0.99 (0.93–1.06)	0.832	1.03 (0.98–1.08)	0.269	0.99 (0.96–1.03)	0.648	1.01×10^−4^
BMAL1	rs7950226	A/G	0.46	1.04 (0.95–1.15)	0.406	1.02 (0.97–1.07)	0.471	0.99 (0.92–1.07)	0.785	1.01 (0.98–1.05)	0.451	0.700
PER1	rs885747	C/G	0.29	0.96 (0.86–1.07)	0.492	1.03 (0.96–1.10)	0.456	NA	NA	1.01 (0.95–1.07)	0.813	0.328
NPAS2	rs895521	T/C	0.15	0.91 (0.80–1.04)	0.184	1.00 (0.94–1.08)	0.875	0.97 (0.92–1.03)	0.327	0.98 (0.94–1.02)	0.294	0.439

MAF – minor allele frequency in UKADS/DGP normoglycaemic control subjects, OR (95% CI) – allelic odds ratio with 95% confidence interval, p – significance level for disease association (p values less than 0.05 are shown in bold), p_het_ – significance level of heterogeneity of odds ratios between datasets, NA – data not available, SNP failed QC in meta-analysis.

Two of the variants were associated with type 2 diabetes in the UKADS/DGP cohort at a study-wide significant level (Table 1). The minor allele (T) of *BMAL1* rs11022775 was associated with susceptibility to the disease (p = 0.003). The rs11022775T/rs7950226A *BMAL1* haplotype was also nominally associated with an increased risk of diabetes in this cohort before correction for multiple testing (p = 0.007); this effect appeared to be driven by the rs11022775 SNP. In contrast, the minor allele (G) of rs7602358 near the *PER2* locus appeared to confer protection against type 2 diabetes in both the UKADS (p = 0.003) and DGP (p = 0.004) datasets (combined cohort, p = 3.18×10^−5^). Neither of these observations was confirmed in the SAT2D or DIAGRAM+ datasets, however. The *BMAL1* rs11022775 SNP was nominally associated with disease in the DIAGRAM+ cohort, but disease risk appeared to be conferred by the C allele, not T as seen in UKADS/DGP. No evidence of an association with rs7602358 was seen in the DIAGRAM+ or SAT2D datasets. In the latter cohort, two *NPAS2* SNPs (rs1369481 and rs17024926) were associated with type 2 diabetes, the former with a p value of 3.78×10^−4^, but this was not replicated in the UKADS/DGP or DIAGRAM+ datasets (Table 1).

**Figure 1:**
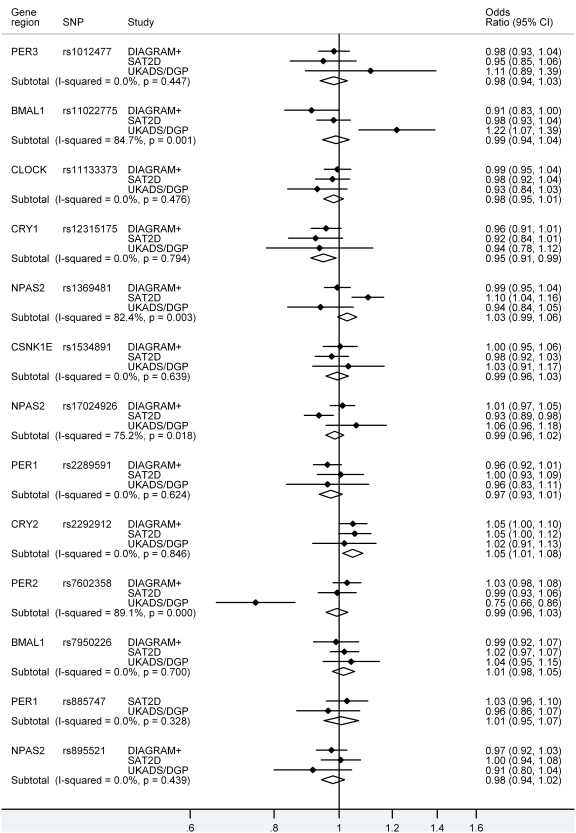
Association of 13 circadian gene SNPs with type 2 diabetes. Forest plot showing the association of 13 circadian gene SNPs with type 2 diabetes in South Asian (UKADS/DGP and SAT2D) and white European (DIAGRAM+) cohorts.

Meta-analysis of all the datasets failed to confirm the disease associations with the *BMAL1* and *PER2* variants. In contrast, type 2 diabetes was associated with rs12315175, close to the *CRY1* gene, and rs2292912, located in the *CRY2* gene (Table 1 and Figure 1), although statistical significance was nominal in both cases.

## Discussion

The pilot study of 13 circadian gene variants in two populations of Punjabi ancestry suggested a significantly protective influence of the G allele of the rs7602358 SNP, located upstream of the *PER2* locus (OR = 0.75, p = 3.18×10^−5^). The association was consistent in independently ascertained indigenous and migrant populations, with almost identical effect sizes (DGP, OR = 0.77; UKADS, OR = 0.73), and was observed in both males and females (Table S2). Furthermore this SNP was more strongly associated with type 2 diabetes in the combined UKADS/DGP cohorts than most of the currently validated type 2 diabetes risk determinants, with the exception of the *TCF7L2* rs7902346 variant [26], leading us to believe that the association might be genuine. This was not confirmed by the analysis of the DIAGRAM+ and SAT2D cohorts, however, which showed no evidence of a role for rs7602358 in disease risk. These findings suggest that we have either identified a false positive association or picked up a variant with a population-specific effect. It should be noted that the South Asians included in the SAT2D study were from different ethnic subgroups to those in the UKADS/DGP cohorts and this could contribute to the discrepancy between the observed effect sizes. The inconsistency in results could not be attributed to differences in minor allele frequency for rs7602358 as it was similar in all the datasets (Table S3).

The *BMAL1* locus was associated with type 2 diabetes in our Punjabi populations, albeit with borderline significance. Our findings differed from those reported previously by Woon *et al* [12], who showed that the rs7950226A/rs11022775C haplotype was associated with an increased risk of diabetes in British families, with the former variant independently more strongly associated than the latter. In contrast, disease susceptibility in the UKADS/DGP cohort was associated with rs7950226A/rs11022775T and this effect appeared to be mediated entirely through rs11022775T. The borderline associations with *BMAL1* variants seen in the UKADS/DGP and DIAGRAM+ datasets and the inconsistency between the results suggest that they are likely to be false positives, a conclusion borne out by the lack of association with either SNP in the meta-analysis.

The analysis of the SAT2D data showed two potentially interesting associations with the *NPAS2* locus, with the rs1369481 variant achieving a reasonable level of significance (p = 3.78×10^−4^). As for the SNPs described above, however, this was not replicated in either of the other datasets and no consistent direction of effect was observed.

Although meta-analysis of the datasets did not confirm disease associations with the *PER2* and *BMAL1* variants, it did provide nominal evidence of associations with *CRY2* rs2292912 and *CRY1* rs12315175. The former SNP is located ∼4.6 kb from rs11605924, which was reported to be associated with type 2 diabetes in the MAGIC study (p = 1.7×10^−4^)[9], with a similar effect size (OR = 1.04) to that seen for rs2292912 in the current meta-analysis (OR = 1.05). These two variants are not in strong linkage disequilibrium (LD) (r^2^ = 0.3 in HapMap Data Release 27; www.hapmap.ncbi.nlm.nih.gov), suggesting that there might be two independent association signals for type 2 diabetes at this locus. The association with rs12315175 near *CRY1* is a novel finding. Interestingly recent studies in mice have suggested a role for Cry1 in glucose homeostasis; hepatic overexpression of the protein was shown to lower blood glucose concentrations and improve insulin sensitivity in insulin-resistant db/db mice [30], while transgenic mice expressing a mutant form of Cry1 developed hyperglycaemia associated with an early-onset insulin-secretory defect [31]. The role of CRY1 in human diabetes may therefore warrant further investigation.

It is interesting to note that the variants displaying significant disease associations in individual populations in our study appear to have opposite directions of effect on type 2 diabetes and prostate cancer; that is the minor alleles of the *CRY2* and *NPAS2* variants that appear to increase the risk of diabetes in one or more of our datasets are associated with a decreased risk of prostate cancer in the study of Zhu *et al* [23], while the putative diabetes-protective alleles near *PER2* and *CRY1* appear to confer an increased risk of prostate cancer. These findings are consistent with the inverse disease relationship reported by meta-analysis of epidemiological data [16]. It should be noted, however, that none of the prostate cancer associations described by Zhu *et al* [23] achieved genome-wide significance and the variants are yet to be confirmed as genuine risk determinants for the disease.

As our pilot study investigated only selected circadian variants, we cannot exclude the possibility that other SNPs at these loci have an influence on disease susceptibility. It is unlikely that such an influence would be major, however, as we would expect this to have been picked up by the genome-wide association analysis of the DIAGRAM+ and SAT2D datasets [27], [28]. Nevertheless it is possible that variants of these genes have a more modest effect, which did not reach the threshold for follow-up in these studies.

In conclusion our study has confirmed the association between type 2 diabetes and variants of the *CRY2* gene and suggested a potential role for the *CRY1* gene in disease development. Together with previous reports of associations between fasting glucose/diabetes and the *MTNR1B* and *CRY2* loci [8], [9], our data support a role for the circadian clock in the regulation of glucose homeostasis.

## Supporting Information

Table S1 **Clinical characteristics of subjects stratified by study population, disease status and sex.**
(DOC)

Table S2 **Sex-specific analysis of circadian gene variants in UKADS/DGP cohort.**
(DOC)

Table S3 **Minor allele frequencies of circadian SNPs in UKADS/DGP, DIAGRAM + and SAT2D datasets.**
(DOC)
