# Supplementary material for: Circadian Gene Variants and Susceptibility to Type 2 Diabetes: A Pilot Study
Source: PLoS One. 2012 Apr 2;7(4):e32670. doi: 10.1371/journal.pone.0032670 (PMC3317653; doi:10.1371/journal.pone.0032670)
Supplement: Table S1 — Clinical characteristics of subjects stratified by study population, disease status and sex. (DOC) [file pone.0032670.s001.doc]

Supporting Information Table S1 - Clinical characteristics of subjects stratified by study population, disease status and sex

|  | **UKADS** | | | | | | | | | | | **DGP** | | | | | | | | | |
| --- | --- | --- | --- | --- | --- | --- | --- | --- | --- | --- | --- | --- | --- | --- | --- | --- | --- | --- | --- | --- | --- |
|  | **NGC** | | | | | **T2D** | | | | | | **NGC** | | | | **T2D** | | | | | |
|  | Female (N=241) | | | Male (N=230) | | Female (N=402) | | | Male (N=490) | | | Female (N=692) | | Male (N=617) | | Female (N=445) | | | Male (N=395) | | |
| Age (years) | 53.1 | (11.7) | | 57.1 | (11.7) | 56.9 | | (12.2) | 57.2 | | (11.9) | 55.4 | (10.5) | 57.6 | (11.2) | 53.7 | (11.3) | | 55.9 | (12.1) | |
| Systolic Blood Pressure (mm Hg) | 131.1 | (20.2) | | 139.5 | (19.6) | 139.1 | | (22.5) | 141.4 | | (19.2) | 130.4 | (20.9) | 126.1 | (19.6) | 136.6 | (21.1) | | 130.9 | (19.9) | |
| Diastolic Blood Pressure (mm Hg) | 82.9 | (12.1) | | 86.0 | (11.6) | 83.7 | | (10.7) | 83.8 | | (11.3) | 81.9 | (13.0) | 80.5 | (12.1) | 85.4 | (11.6) | | 83.6 | (11.6) | |
| Total cholesterol  (mmol/l) | ND | | | ND | | 4.84 | | (1.17) | 4.69 | | (1.15) | 4.72 | (1.17) | 4.71 | (1.07) | 4.98 | (1.19) | | 4.71 | (1.05) | |
| LDL cholesterol  (mmol/l) | ND | | | ND | | 2.44 | | (0.94) | 2.43 | | (1.01) | 2.89 | (1.11) | 2.90 | (0.99) | 2.96 | (1.14) | | 2.75 | (1.03) | |
| HDL cholesterol  (mmol/l) | ND | | | ND | | 1.34 | | (0.40) | 1.26 | | (0.54) | 1.20 | (0.30) | 1.11 | (0.29) | 1.09 | (0.31) | | 1.00 | (0.31) | |
| Triglycerides (mmol/l) | ND | | | ND | | 2.71 | (2.38) | | 2.59 | (1.80) | | 1.64 | (1.03) | 1.83 | (1.06) | 2.34 | | (1.51) | 2.37 | | (1.44) |
| Blood glucose (mmol/l) | 5.41a | | (0.90) | 5.33a | (0. 89) | ND | | | ND | | | 4.73b | (0.48) | 4.78b | (0.47) | ND | | | ND | | |
| HbA1c­ (%) | ND | | | ND | | 8.27 | | (1.72) | 8.32 | | (1.98) | ND | | ND | | 9.70 | (3.28) | | 9.58 | (3.16) | |
| BMI (kg/m2) | 28.9 | (5.3) | | 27.2 | (4.1) | 30.0 | | (4.9) | 27.3 | | (3.9) | 24. 9 | (5.3) | 23.6 | (4.6) | 26.7 | (5.0) | | 25.4 | (4.2) | |
| Waist circumference (cm) | 99.1 | (12.2) | | 100.8 | (10.8) | 104.0 | | (10.7) | 100.8 | | (10.0) | 92.5 | (13.9) | 91.5 | (11.8) | 97.2 | (12.1) | | 96.4 | (11.7) | |
| Height (m) | 1.58 | (0.07) | | 1.70 | (0.07) | 1.57 | | (0.06) | 1.71 | | (0.07) | 1.57 | (0.09) | 1.70 | (0.08) | 1.57 | (0.08) | | 1.70 | (0.09) | |
| Weight (kg) | 71.8 | (14.1) | | 78.4 | (12.6) | 73.8 | | (13.3) | 79.6 | | (12.3) | 61.4 | (13.6) | 68.5 | (14.0) | 65.6 | (12.4) | | 73.8 | (13.3) | |

All values are means (SD). NGC = normoglycaemic control subjects. T2D = type 2 diabetes. ND = not determined. a = random blood glucose. b = fasting blood glucose. Within the UKADS NGC group phenotypic data were available for a maximum of 285 subjects, with the exception of age which was available for all subjects.
