# Supplementary material for: Circadian Gene Variants and Susceptibility to Type 2 Diabetes: A Pilot Study
Source: PLoS One. 2012 Apr 2;7(4):e32670. doi: 10.1371/journal.pone.0032670 (PMC3317653; doi:10.1371/journal.pone.0032670)
Supplement: Table S2 — Sex-specific analysis of circadian gene variants in UKADS/DGP cohort. (DOC) [file pone.0032670.s002.doc]

# Supporting Information Table S2 – Sex-specific analysis of circadian gene variants in UKADS/DGP cohort

|  |  | Females | | | | | Males | | | | |  |
| --- | --- | --- | --- | --- | --- | --- | --- | --- | --- | --- | --- | --- |
| Gene region | SNP | OR | 95% CI | SE | z | p | OR | 95% CI | SE | z | p | p_het_ |
| PER3 | rs1012477 | 1.032 | 0.751-1.420 | 0.168 | 0.2 | 0.845 | 1.221 | 0.890-1.677 | 0.198 | 1.24 | 0.216 | 0.463 |
| BMAL1 | rs11022775 | 1.127 | 0.955-1.329 | 0.095 | 1.42 | 0.156 | 1.232 | 1.043-1.455 | 0.105 | 2.46 | 0.014 | 0.454 |
| CLOCK | rs11133373 | 0.836 | 0.722-0.968 | 0.063 | -2.39 | **0.017** | 1.026 | 0.890-1.183 | 0.075 | 0.35 | 0.724 | 0.050 |
| CRY1 | rs12315175 | 0.929 | 0.719-1.200 | 0.121 | -0.57 | 0.572 | 0.953 | 0.734-1.237 | 0.127 | -0.36 | 0.718 | 0.890 |
| NPAS2 | rs1369481 | 1.064 | 0.907-1.248 | 0.087 | 0.76 | 0.445 | 0.826 | 0.705-0.969 | 0.067 | -2.35 | **0.019** | 0.028 |
| CSNK1E | rs1534891 | 1.108 | 0.935-1.313 | 0.096 | 1.18 | 0.238 | 0.949 | 0.795-1.134 | 0.086 | -0.58 | 0.565 | 0.218 |
| NPAS2 | rs17024926 | 1.005 | 0.869-1.161 | 0.074 | 0.06 | 0.952 | 1.127 | 0.971-1.307 | 0.085 | 1.58 | 0.115 | 0.277 |
| PER1 | rs2289591 | 1.024 | 0.838-1.252 | 0.105 | 0.23 | 0.815 | 0.906 | 0.743-1.106 | 0.092 | -0.97 | 0.332 | 0.369 |
| CRY2 | rs2292912 | 1.044 | 0.899-1.213 | 0.798 | 0.56 | 0.572 | 0.991 | 0.845-1.161 | 0.080 | -0.12 | 0.907 | 0.636 |
| PER2 | rs7602358 | 0.785 | 0.665-0.928 | 0.067 | -2.83 | **0.005** | 0.782 | 0.658-0.929 | 0.069 | -2.79 | **0.005** | 0.973 |
| BMAL1 | rs7950226 | 0.990 | 0.865-1.133 | 0.068 | -0.14 | 0.885 | 1.097 | 0.954-1.261 | 0.078 | 1.30 | 0.195 | 0.301 |
| PER1 | rs885747 | 0.944 | 0.813-1.096 | 0.072 | -0.76 | 0.448 | 0.982 | 0.840-1.148 | 0.078 | -0.23 | 0.820 | 0.719 |
| NPAS2 | rs895521 | 0.829 | 0.687-1.003 | 0.080 | -1.93 | 0.054 | 1.010 | 0.837-1.220 | 0.097 | 0.11 | 0.915 | 0.149 |

# OR – allelic odds ratio for type 2 diabetes; 95% CI – 95% confidence interval; SE – standard error; z – z-score; p – significance level for disease association (p values less than 0.05 are shown in bold), p_het_ – significance level of heterogeneity of odds ratios between males and females.

The OR values did not differ significantly between males and females after correction for multiple testing.
