# Supplementary material for: Circadian Gene Variants and Susceptibility to Type 2 Diabetes: A Pilot Study
Source: PLoS One. 2012 Apr 2;7(4):e32670. doi: 10.1371/journal.pone.0032670 (PMC3317653; doi:10.1371/journal.pone.0032670)
Supplement: Table S3 — Minor allele frequencies of circadian SNPs in UKADS/DGP, DIAGRAM + and SAT2D datasets. (DOC) [file pone.0032670.s003.doc]

# Supporting Information Table S3. Minor allele frequencies of circadian SNPs in UKADS/ DGP, DIAGRAM+ and SAT2D datasets.

|  |  |  | UKADS/DGP | SAT2D | DIAGRAM+ |
| --- | --- | --- | --- | --- | --- |
| Gene region | SNP | Allele  (minor/common) | MAF | MAF | MAF |
| PER3 | rs1012477 | C/G | 0.05 | 0.05 | 0.14-0.24 |
| BMAL1 | rs11022775 | T/C | 0.16 | 0.19 | 0.02-0.09 |
| CLOCK | rs11133373 | G/C | 0.38 | 0.4 | 0.25-0.36 |
| CRY1 | rs12315175 | C/T | 0.07 | 0.06 | 0.18-0.23 |
| NPAS2 | rs1369481 | T/C | 0.24 | 0.23 | 0.18-0.33 |
| CSNK1E | rs1534891 | T/C | 0.19 | 0.19 | 0.11-0.18 |
| NPAS2 | rs17024926 | C/T | 0.32 | 0.29 | 0.28-0.49 |
| PER1 | rs2289591 | A/C | 0.14 | 0.17 | 0.21-0.27 |
| CRY2 | rs2292912 | C/G | 0.27 | 0.25 | 0.20-0.26 |
| PER2 | rs7602358 | G/T | 0.16 | 0.15 | 0.17-0.26 |
| BMAL1 | rs7950226 | A/G | 0.46 | 0.5 | 0.42-0.49 |
| PER1 | rs885747 | C/G | 0.29 | 0.23 | NA |
| NPAS2 | rs895521 | T/C | 0.15 | 0.14 | 0.18-0.24 |

# MAF- minor allele frequency in normoglycaemic control subjects, SAT2D MAF estimates are taken from the LOLIPOP samples (see reference [28]); NA – data not available, SNP failed QC in meta-analysis
